# Rates and correlates of cannabis-associated psychotic symptoms in over 230,000 people who use cannabis

**DOI:** 10.1038/s41398-022-02112-8

**Published:** 2022-09-06

**Authors:** Tabea Schoeler, Jason Ferris, Adam R. Winstock

**Affiliations:** 1 Department of Computational Biology, University of Lausanne, Lausanne, Switzerland; 2 Clinical, Educational and Health Psychology, University College London, London, UK; 3 The Centre for Health Services Research, The University of Queensland, Brisbane, QLD Australia; 4 Institute of Epidemiology and Health Care, University College London, London, UK; 5 Global Drug Survey, GDS, London, UK

**Keywords:** Schizophrenia, Addiction

## Abstract

Cannabis, a widely used psychoactive substance, can trigger acute cannabis-associated psychotic symptoms (CAPS) in people who use cannabis (PWUC). To assess rates and correlates of CAPS requiring emergency medical treatment, we analyzed data from an international sample of PWUC (*n* = 233,475). We found that 0.47% (95%CI 0.42; 0.52) PWUC reported lifetime occurrence of CAPS, defined as the occurrence of hallucinations and/or paranoia requiring emergency medical treatment following the use of cannabis. A range of factors correlated with risk of CAPS in the last year: higher rates were observed in young individuals [risk ratio (RR) 2.66, compared to older PWUC] and those residing in Denmark (RR 3.01, compared to PWUC from other countries). Furthermore, risk was elevated in those using predominantly high-potency resin (RR 2.11, compared to PWUC using herbal cannabis), those mixing cannabis with tobacco (RR 2.15, compared to PWUC not mixing with tobacco) and those with a diagnosis of psychosis (RR 14.01), bipolar (RR 4.30), anxiety (RR 2.92) and depression (RR 2.68), compared to individuals without a mental health diagnosis. Taken together, acute self-limiting psychotic symptoms in the context of cannabis use may occur in about 1 in 200 PWUC’s lifetime. Some individuals could be particularly sensitive to the adverse psychological effects of cannabis, such as young individuals or those with pre-existing mental health vulnerabilities. In light of the movements towards legalization of recreational cannabis, more research should focus on the potential harms related to cannabis use, to educate PWUC and the public about risks related to its use.

## Introduction

Cannabis is one of the most commonly used psychoactive substance in Europe [1] and across the world [2], with about one in four adults having tried cannabis during their lifetime [1]. People who use cannabis (PWUC) report to consume cannabis because of its acute psychoactive effects, including the “high” that is responsible for the pleasant experiences such as relaxation, euphoria or sociability [3]. Given the epidemiological evidence linking cannabis use to long-term risk of psychosis [4, 5] and the emergence of high-potency forms of cannabis in recent years [6, 7], there is now growing concern about the potential adverse effects of recreational cannabis, particularly in young individuals [5, 8]. Acute adverse reactions following cannabis use can include transient undesired psychiatric effects, such as anxiety, panic or psychosis-like experiences involving hallucinations or paranoia [9, 10]. The documented psychotomimetic effects are largely attributable to THC, the main psychoactive compound in cannabis, which acts on the central nervous system by primarily binding on the CB_1_ cannabinoid receptors [11]. It has been shown that transient cannabis-associated psychosis-like experiences occur in some PWUC, involving experiences such as paranoia (reported by between 15% and 53% [10, 12–19] of PWUC) or hallucinations (reported by between 3% and 27% [13–16, 19, 20] of PWUC) during cannabis intoxication. Although less common, some PWUC experience severe cannabis-associated psychosis requiring medical attention [21].

Given the significant percentage of individuals experiencing psychosis-like symptoms while under the influence of cannabis, and with the current move towards legalization of cannabis for medical and recreational purposes in numerous countries, increasing attention is now being paid to the acute [10, 22] and longer term psychiatric effects, notably risk for psychosis [23, 24]. Of particular interest is the question as to what characteristics are common among people who are most sensitive to its psychotomimetic effects [25]. Controlled experiments [26] have shown that the severity of the undesirable experiences following THC administration varies within samples, implicating that individual-level factors may contribute to variations in cannabis-associated psychotomimetic experiences. For example, it has been implicated that young individuals and those with pre-existing mental health vulnerabilities are at an increased risk of adverse experiences when using cannabis [15, 27]. Together with evidence highlighting the role of cannabis use characteristics (e.g., dose of cannabis, level of tolerance) in modulating THC-effects [28–30], risk of adverse experiences following cannabis use may be composed of an interplay between individual-level factors (e.g., pre-existing mental health) and cannabis use characteristics.

Despite extensive research into the nature of cannabis intoxication, evidence is still lacking with regard to rates and correlates of cannabis-associated psychotic symptoms (referred to as CAPS hereafter) warranting clinical attention, such as events requiring emergency medical treatment [31–33] due to the emergence of psychotic symptoms following cannabis use. Thus far, only few studies have specifically focused on rates of CAPS requiring medical treatment [21, 31–33]. Most published studies have more broadly assessed substance-induced outcomes [34–37], without restricting the sample to cannabis-exposed individuals (cf. Supplementary Table S1 for an overview). Furthermore, while existing studies have typically focused on assessed rates and predictors of subclinical psychotic-like experiences [9, 15, 16, 20, 25, 38–40], there has been little work on CAPS requiring medical attention. A systematic investigation on rates of CAPS severe enough to warrant acute medical assessment in a large sample of PWUC is therefore needed, to derive estimates that generalize specifically to cannabis using individuals. Moreover, important predictors of CAPS are typically not assessed in registry-based studies, such as individual characteristics that could plausibly link to CAPS (e.g., frequency, quantity or duration of cannabis use [25], type of cannabis used [41]). Exploring individual differences influencing risk of CAPS is key to advancing our understanding of the link between cannabis use and mental health, for a number of reasons. First, such knowledge will help to identify and safeguard those individuals that are at highest risk of triggering episodes of CAPS when using cannabis. Second, individuals with cannabis-induced psychosis are at high risk of converting to a psychotic disorder in the long-term [21, 42, 43], in line with evidence implicating cannabis in the etiology of psychosis [44–47]. As such, a better understanding of factors predicting CAPS has the potential to inform prevention of acute adverse events and the development of psychosis in the context of cannabis use. Finally, a number of factors have previously been implicated in the emergence of transient symptoms of CAPS, notably psychosis-liability [15, 22, 48, 49], cannabis potency [41] and young age [14]. Assessing if aforementioned factors also link to risk of severe episodes of CAPS would therefore validate previous lines of evidence and provide further support for their clinical relevance when trying to identify vulnerable individuals.

In summary, a comprehensive investigation on rates and correlates of cannabis-associated psychotic symptoms requiring emergency medical treatment will bolster the evidence base on the complex relationship between cannabis use and mental health. More specifically, our study aims to examine three main questions:

1. What are the rates of CAPS requiring emergency medical treatment (or assessment) in a large international sample of PWUC?
2. Do rates of CAPS vary as a function of demographic characteristics (e.g., country of residence, age, gender), cannabis/substance use patterns (e.g., type of cannabis used, route of administration, other substance use) and individual vulnerabilities (e.g., mental health)?
3. Characterization of PWUC with CAPS, including the patterns of use of cannabis and other substances just before seeking emergency medical treatment due to the occurrence of psychotic symptoms.

## Methods

### Sample

Data was drawn from the largest online drug survey world-wide, the Global Drug Survey (GDS) [50], including data from five years of GDS data collection (2014, 2015, 2016, 2017, 2019). Out of the total number of participants taking part in the five GDS years (*N* = 529,574), we selected *n* = 233,475 individuals, including those (1) with a history cannabis use, (2) who resided in a participating country that had a response rate of at least *n* = 1000 PWUC and (3) had complete data on CAPS. A more detailed description of the GDS procedures can be found in the Supplement. Ethical approval was received from The Psychiatry, Nursing and Midwives Ethics subcommittee at Kings College, London (141/02), The University of Queensland (No: 2017001452) and The University of New South Wales (HREC HC17769). All respondents provided informed consent.

### Measures

The main outcome of our study—cannabis-associated psychotic symptoms (CAPS) requiring emergency medical treatment—was assessed using two items screening for cannabis-associated emergency medical treatment, experienced either in the last year (“In the past 12 month, have you sought emergency medical treatment following your use of cannabis?) or per lifetime (“Have you ever sought emergency medical treatment following your use of cannabis?”). Those individuals reporting cannabis-associated emergency medical treatment were asked to endorse from a list of 19 physical and psychological items their symptom presentation (cf., Box 1, Supplement). Individuals who reported to have sought emergency medical treatment due to the occurrence of psychotic symptoms following cannabis use, including hallucinations (“Seeing, hearing things”) and paranoia (“Paranoia, suspiciousness”) were then classified as PWUC with CAPS. To assess correlates of CAPS, we used data from a number of questions assessing factors that could plausibly link to cannabis-psychosis, such as age, mental health history (“Have you ever been diagnosed with a mental illness?”), type of preferred cannabis (“Over the last 12 months, which type of cannabis have used most commonly?”), route of administration (“Which is the most common way you currently use cannabis?”), frequency of cannabis use (“How many days have you used cannabis in the last 12 months?”), among others. Details regarding the assessment and coding of all included measures can be found in the sMethods (Supplement) and Supplementary Table S2. Finally, we analyzed data from items characterizing the CAPS event and its consequences (e.g., type and amount of cannabis used before seeking to emergency medical treatment for CAPS).

### Statistical analyses

All analyses were conducted in R version 4.0 [51]. First, rates were estimated separately for PWUC with data on last year (*n* = 148,109) or lifetime (*n* = 85,366) occurrence of CAPS requiring emergency medical treatment. The corresponding confidence intervals (95% CI) of rates of CAPS were derived using the R package PropCIs [52], which uses the Clopper-Pearson exact method [53]. Second, to examine correlates of CAPS, we estimated rates of CAPS in subgroups of PWUC, stratified according to a number of factors that could plausibly link to CAPS (e.g., gender, age, cannabis/substance use characteristics, mental health diagnoses). In this analysis, only the sub-sample with data on last year CAPS was included (*n* = 148,109). To quantify the magnitude of differences in rates of CAPS between subgroups, we estimated risk ratios and the corresponding 95% CI and *p*-values as implemented in the R package fmsb [54]. Given the substantial number of conducted tests, false discovery rate (FDR) was controlled using the Benjamin and Hochberg FDR-correction method [55]. Of note, comparisons of rates of CAPS across subsets of PWUC were only interpreted if each subset included at least 15 individuals with CAPS. This was done to ensure accurate estimation of rates of CAPS [56]. Nevertheless, for the interested reader, the full set of estimates is reported in the Supplement. Finally, since the sample used for the main analyses also included a number of individuals who have taken part in previous GDS years, we carried out sensitivity analyses to assess risk of bias due to repeated participation. Here, rates of CAPS were estimated only in those PWUC reporting to have not taken part in pervious GDS years.

## Results

### Rates of cannabis-associated psychotic symptoms requiring emergency medical treatment

**Fig. 1:**
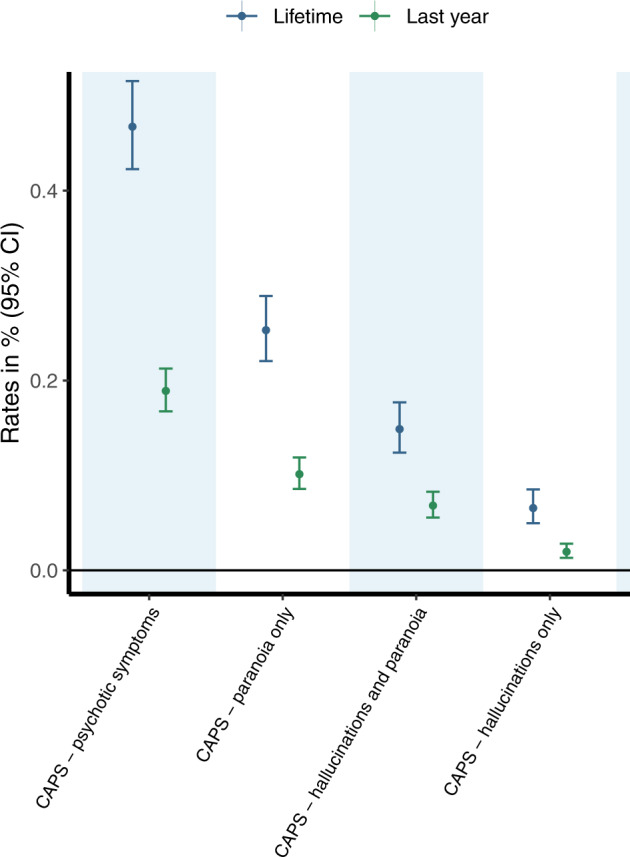
Rates of lifetime and last year cannabis-associated psychotic symptoms (CAPS) requiring emergency medical treatment. Estimated are rates of cannabis-associated psychotic symptoms (CAPS) resulting in emergency medical treatment in people who use cannabis (PWUC). Rates are plotted separately for lifetime risk of CAPS (*n* = 85,366, displayed in blue) and last year risk of CAPS (*n* = 148,109, displayed in green). The legend refers to the different symptom profiles of CAPS leading to emergency medical treatment, including the occurrence of either hallucinations or paranoia (“CAPS—psychotic symptoms”), hallucinations and paranoia (“CAPS—hallucinations and paranoia”), only paranoia but no hallucinations (“CAPS—paranoia only”), or only hallucinations but no paranoia (“CAPS—hallucinations only”). The proportion of PWUC reporting to have sought emergency medical treatment following cannabis use (not limited to psychotic symptoms only) included 1.11% (1.04; 1.19) and 0.59 (0.55; 0.63) in the lifetime and in the last year, respectively. All corresponding estimates are provided in Supplementary Table S3.

A summary of the sample characteristics is provided in the sResults (Supplement) and Supplementary Table S3. As shown in Fig. 1 and reported Supplementary Table S4, an estimated 0.47% (95%CI 0.42; 0.52) of PWUC sought emergency medical treatment during their lifetime due to the occurrence of CAPS following cannabis use. In the last year, 0.19% (95%CI 0.17; 0.21) of PWUC reported CAPS requiring emergency medical treatment following cannabis use. CAPS involving paranoia only (last year: 0.1%; lifetime: 0.25%) was the most commonly reported symptom profile, followed by CAPS involving both hallucinations and paranoia (last year: 0.07%; lifetime: 0.15%). Least common was CAPS involving hallucinations only (last year: 0.02%; lifetime: 0.07%). As a sensitivity analysis, rates of CAPS were also estimated in a sub-sample excluding individuals who have indicated to have taken part in a previous GDS survey. Rates of CAPS in this sub-sample (shown in Supplementary Table S5) were comparable to those estimated for all included individuals, implicating that sample overlap is unlikely to have biased our estimates.

### Correlates of cannabis-associated psychotic symptoms requiring emergency medical treatment

**Fig. 2:**
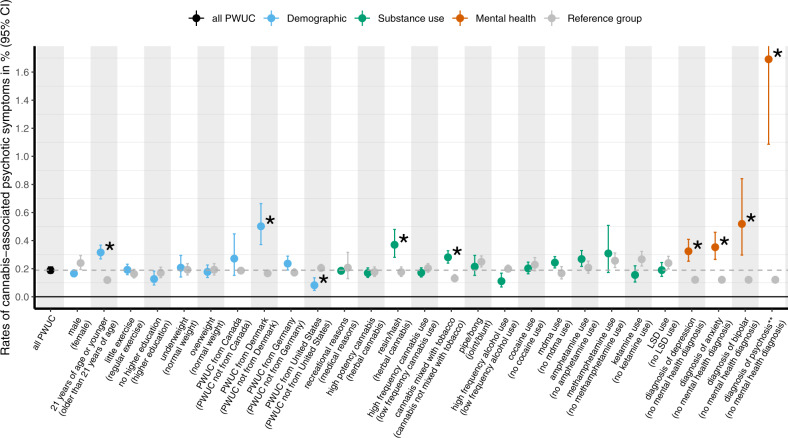
Rates of cannabis-associated psychotic symptoms (CAPS) in subgroups of people who use cannabis (PWUC). Rates are estimated for cannabis-associated psychotic symptoms (CAPS, involving hallucinations and/or paranoia) requiring emergency treatment in subgroups of people who use cannabis (PWUC). The sample was restricted to individuals with data on last year CAPS (*n* = 148,109). The dashed line (gray) indicates the overall rate of last year CAPS (i.e., 0.19%, also displayed in Fig. 1). Of all subgroup comparisons (*k* = 53), displayed here are only those conducted in subgroups including at least 15 individuals with documented last year CAPS. The complete table of all subgroup comparisons, as well as the corresponding Risk Ratios, is included in Supplementary Table S6. Detailed information regarding the coding of the grouping variables is provided in the sMethods (Supplement). The asterisk (*) highlights those comparisons that were significantly different (*p* < 0.05) in univariate analyses after False Discovery Rate (FDR) correction to adjust for multiple testing (*k* = 53 comparisons). **Subgroup of PWUC who reported to have been diagnosed with psychosis excludes those individuals with CAPS that did not return to normal within 4 weeks following the event.

As shown in Fig. 2 and in Supplementary Table S2/Supplementary Table S6, a number of factors were significantly associated with risk of CAPS in the last year, after correcting for multiple testing. First, with respect to demographic factors, significantly higher rates were present in PWUC below the age of 21 when compared to older individuals (Relative Risk, RR = 2.66). Rates of CAPS were also higher in PWUC residing in Denmark (RR = 3.01), when compared to the rest of the sample. Post-hoc exploratory analyses indicated that the elevated rates of CAPS in Denmark may reflect the popularity of high-potency resin in this country (cf. sResults in Supplement and Supplementary Table S7 for detailed results). Lower rates of CAPS were present in PWUC residing in the United States (RR = 0.4). However, we found that rates of CAPS were higher in participants mixing cannabis with tobacco when compared to those consuming cannabis without tobacco (RR = 2.15). With respect to cannabis use characteristics, risk of CAPS was not significantly increased in PWUC reporting frequent use of cannabis or in those using mostly high-potency cannabis (e.g., skunk) in the previous year. Similarly, the route of administration (e.g., bong, joint) and the use of other psychoactive substances (e.g., alcohol, MDMA, cocaine, amphetamines, Ketamine, LSD) in the previous year did not significantly elevate risk of CAPS.

Finally, having a mental health diagnosis was linked to higher rates of CAPS. The highest risk estimates were present in users with a psychotic disorder (RR = 14.01, absolute risk = 1.69%), followed by those with a diagnosis of bipolar (RR 4.30), anxiety (RR = 2.92) and depression (RR = 2.68). All estimates of absolute risk, including the corresponding confidence intervals, are provided in Supplementary Table S6.

### Characterization of individuals with cannabis-associated psychotic symptoms requiring emergency medical treatment

**Fig. 3:**
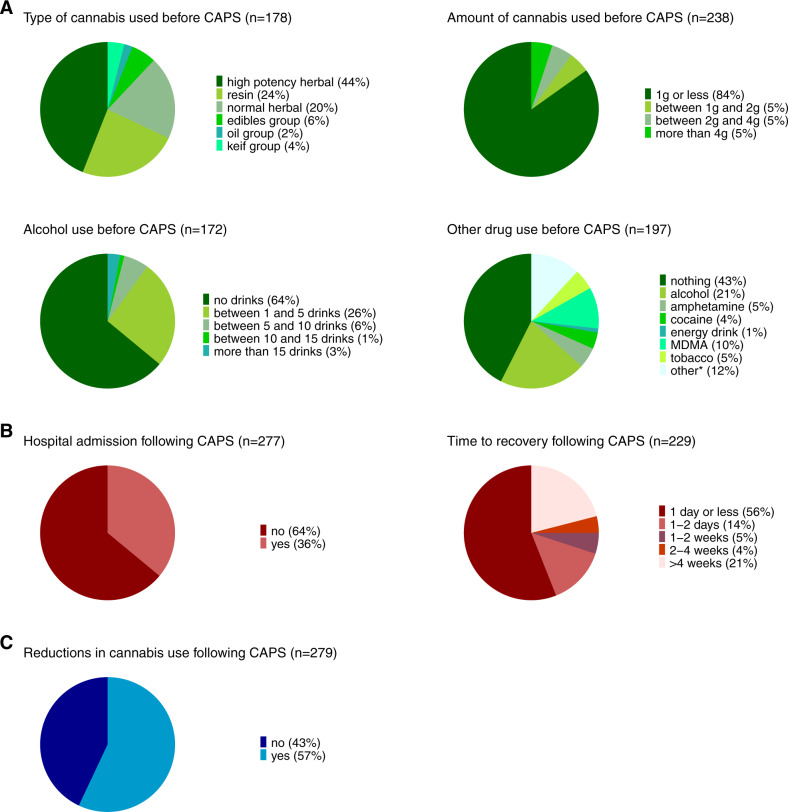
Characterization of people who use cannabis with cannabis-associated psychotic symptoms (CAPS). Results are plotted for people who use cannabis (PWUC) with last year cannabis-associated psychotic symptoms (CAPS) leading to emergency medical treatment. Depicted are **A** the surrounding circumstances before the occurrence of CAPS, **B** information on treatment and recovery of CAPS and **C** reports on changes in cannabis use following the occurrence of CAPS. All corresponding estimates are provided in Supplementary Table 9, including estimates characterizing PWUC with lifetime CAPS (not plotted here). *Includes all other substances that were reported by 10 or less PWUC with CAPS.

Figure 3 illustrates the information gathered around the circumstances surrounding the occurrence of CAPS in those seeking emergency medical treatment in the last year. As highlighted in green (Fig. 3A), the most typical pattern describing the circumstances around the event of CAPS was that of using high-potency cannabis (44%) or resin (24%). The majority of cases reported to have used 1 g or less of cannabis (84%). Most individuals with CAPS did not use other drugs before seeking emergency medical treatment (43%), while others reported to have used alcohol (21%) or MDMA (10%). An excessive pattern of drinking (i.e., more than 15 drinks) before seeking emergency medical treatment was, however, rare (3%). While the majority of users returned back to normal within one day or less following the emergence of CAPS (56%), a subset individuals experienced prolonged psychotic symptoms (e.g., longer than 4 weeks: 21%) (cf., Fig. 3B). 36% of individuals seeking emergency medical treatment for CAPS were subsequently admitted to hospital. Additional characterization of individuals requiring hospital admission (Supplementary Table S10) showed that rates of admission were most elevated in individuals with a lifetime diagnosis of psychosis (76%) and in individuals with prolonged CAPS (54%, i.e., individuals only recovering after more than one week). Other characteristics, including age, gender, type of cannabis consumed and symptoms profile of CAPS (hallucinations versus paranoia), were not significantly linked to risk of hospital admission.

Regarding changes in cannabis use following the occurrence of CAPS (cf., Fig. 3C), 43% of cases did not reduce their cannabis use as a result. Post-hoc analyses showed that the majority of individuals reducing their cannabis use were those recovering within a day or less (53%) and individuals not requiring hospital admission (56%). All estimates are reported in Supplementary Table S9.

## Discussion

This study, considered the largest to date, focuses on cannabis-associated psychotic symptoms requiring emergency medical treatment (labeled as CAPS hereafter) in a sample of people who use cannabis (PWUC). Our results highlight that CAPS can occur in a subset of cannabis users and that a number factors are associated with an elevated risk of CAPS (e.g., young age, mental health vulnerabilities, particularly psychosis-liability, the use of high-potency resin). In individuals with lived experience of CAPS, the event was characterized by the use of predominantly high-potency forms of cannabis, and mostly not due to the co-use of other substances. In the next sections, we will discuss in more detail our results with regard to our three main research questions, namely what are (1) the rates, (2) the correlates and (3) the characterizing circumstances of CAPS in a global sample of PWUC.

### Rates of cannabis-associated psychotic symptoms requiring emergency medical treatment

We found that less than one percent (0.5%) of the PWUC included in our study required emergency medical treatment at some point in their life due to the emergence of CAPS. The rates of CAPS found in our study are higher compared to previous studies looking at similar outcomes, including rates of cannabis-induced psychosis as estimated in registry-based samples [21, 57] (also see Supplementary Table S1). Most likely, this discrepancy reflects the fact that previous studies did not restrict their samples to cannabis-exposed individuals only when assessing CAPS, which would deflate risk estimates. In addition, rates of CAPS may be lower in studies conducted at a time when cannabis was less potent than it is today, in line with research showing that the rates of cannabis-induced psychosis have doubled in the past 15 years [58].

Given the significant rate of CAPS in our study (about 1 in 200 PWUC), our results suggest that acute self-limiting psychotic symptoms in the context of cannabis use can occur. While transient psychosis-like symptoms not requiring medical attention may occur in around one third of individuals exposed to cannabis [19, 20], the occurrence of severe acute CAPS leading to emergency medical treatment are less common. Rates of CAPS as observed here are comparable to rates of other drug-induced psychosis, such as alcohol-associated psychosis (around 0.4–0.7% [59]), while rates of stimulant-induced psychosis are likely to be somewhat higher [60].

Together with previous findings implicating CAPS as a marker on the path leading to primary psychosis [21, 43, 61, 62], this study highlight the importance of further research focusing on predictors and long-term consequences of CAPS, to inform public health initiatives and treatment programs to better safeguard vulnerable PWUC.

### Correlates of cannabis-associated psychotic symptoms requiring emergency medical treatment

Given the richness of the data analyzed in this study, in particular with regard to substance and cannabis use characteristics, we were able to explore numerous factors not previously assessed in registry-based samples focusing on CAPS. Concerning demographic characteristics, a number of factors appeared to affect an individual’s propensity to experience CAPS. Notably, risk of CAPS was higher among younger PWUC, in line with previous findings reporting higher rates of cannabis-induced symptoms in younger individuals [14]. This finding is consistent with the idea of developmental sensitivity to the psychotomimetic effects of cannabis [23, 63], implicating that the still maturing brain may be particularly vulnerable to adverse effects of cannabis. When estimating rates of CAPS across countries, we observed the highest rates among PWUC residing in Denmark, where resin was the most popular type of cannabis. As such, the higher rates in Denmark may be attributable to the use of Danish resin, which, with a THC concentration of 23% or higher since 2014 [64], is one of most potent forms of cannabis in Europe. This is in line with our finding showing that rates of CAPS were higher among individuals using mostly hash/resin when compared to those consuming normal herbal cannabis. Considering that the increases in THC concentrations are particularly high for cannabis resin forms in recent years [65], our results suggest that the use resin is now potentially more harmful than it was years ago. Surprisingly, rates of CAPS were lower in the United States. Based on post-hoc analyses, the lower rates in the US are unlikely to reflect the higher costs of care in the US that may prevent people from seeking emergency medical treatment (cf., sDiscussion for more details, Supplement). Of note, the observed differences in rates of CAPS across countries should only be interpreted with caution, as rates of emergency medical treatment following cannabis use may be masked by country-specific factors, such as differences in access to health care services or the level of social tolerance and stigma towards substance use.

Risk of CAPS was also higher in PWUC mixing cannabis with tobacco. While this is in line with research implicating tobacco use in the etiology of psychosis [66, 67], tobacco did not alter the psychosis-like effects induced by cannabis when tested in experimental studies [68]. As such, additional research should further evaluate if tobacco modulates the effects of THC.

We did not find an effect of frequency of cannabis use on CAPS. This is, however, similar to previous observational evidence focusing on cannabis-induced psychosis-like experiences, where cannabis frequency [9, 15], cannabis dependency [38] or duration of cannabis use [15] did not affect outcome. While this seems to contrast experimental findings asserting a dose-response relationship between cannabis and psychosis-like symptoms [69], frequency of cannabis use may be more of a risk marker for the development of primary psychosis over the long-term [47, 70, 71], rather than a factor affecting acute reactivity to cannabis. Instead, frequency of cannabis use in PWUC may be associated with tolerance to the psychotomimetic effects of cannabis, as suggested by experimental studies [72–74].

Finally, with regard to the association between mental health and CAPS, we found that rates of CAPS were higher in those with a diagnosis of bipolar, anxiety or depressive disorder, and highest in individuals with a diagnosis of psychosis. Together with existing evidence linking depression, anxiety and psychosis-liability to cannabis-associated psychosis-like symptoms [15, 20, 22, 48, 49, 75], our findings are in line with the idea of a common (genetic) vulnerability representing risk that is shared across psychiatric disorders [76]. Conversely, the particularly high rates of CAPS in individuals with psychosis may reflect risk stemming from a vulnerability that is specific to the psychotic spectrum. Noteworthy, mental health as assessed in our study is reported as a lifetime diagnosis. As such, we are unable to parse out whether the diagnosis of psychosis (and other mental health diagnoses) preceded the occurrence of CAPS. However, to restrict our analysis to individuals with a pre-existing diagnosis of psychosis only, we excluded PWUC who reported prolonged psychotic symptoms following the occurrence of CAPS.

### Characterization of individuals with cannabis-associated psychotic symptoms requiring emergency medical treatment

Finally, we found that the occurrence of CAPS in PWUC was characterized by a pattern of cannabis use that involved the use of high-potency cannabis forms before seeking emergency medical treatment. This finding suggests that frequency of cannabis use, over stretches of time, may be less of a risk factor for acute psychotic symptoms (as discussed above). Instead, it appears to be the use of high-potency cannabis that carries a substantial risk of CAPS, perhaps in individuals who are otherwise not used to smoking potent forms of cannabis. While the largest proportion of individuals had only used cannabis prior to seeking emergency medical treatment, a third of them had also other psychoactive substances. In particular, alcohol was typically used before seeking emergency medical treatment. An excessive pattern of drinking before CAPS was, however, rare. The majority of individuals returned back to normal within one day or less following CAPS, although a subset reported prolonged psychotic symptoms and required hospital admission. Surprisingly, almost half of the PWUC with CAPS did not reduce their cannabis use as a result of their experience. When treating individuals with cannabis-associated psychotic symptoms in the emergency setting, it is therefore paramount to educate PWUC about the risks associated with the use of cannabis—such advice constitutes a first step towards prevention of adverse cannabis-related outcomes in the future.

## Limitations

This study should be interpreted in the context of three potential methodological limitations. First, it is worth noting that CAPS as assessed in this study capture only cannabis-associated symptoms warranting clinical attention, implicating that the rates of less severe acute psychotic symptoms in cannabis users lie higher than the rates estimated in this study. Second, data on CAPS was obtained retrospectively using self-report measures, raising the question of recall bias and subjective reporting of psychotic symptoms. However, given the severity of the CAPS event (i.e., requiring emergency medical treatment), we believe that the aforementioned biases are unlikely to have affected the validity of our outcome measure. While recall bias could potentially affect the validity of some of the included predictors (e.g., frequency of cannabis use), we had restricted the correlation analyses to variables relating to past year behaviors (e.g., frequency of cannabis use in the last year) only, to minimize the risk of bias. Of note, data was collected using non-representative sampling, which has advantages and disadvantages, including those surrounding reliability and validity at a population-based level, as discussed elsewhere [77–79]. However, the anonymous web survey design employed here is particularly valuable for the collection of data on sensitive topics, including substance use, as such design can provide a sense of privacy that is missing in face-to-face interviews. In addition, our study was set out to analyze data from a large sample restricted to people who report recreational cannabis use. This selected sample has been shown to have similar characteristics to samples of cannabis users produced by probability sampling [80] (cf., Supplement for further discussion). Finally, when testing the association between diagnosis of psychosis and risk of CAPS, our estimate may be underestimated, for two main reasons: (1) individuals who developed prolonged psychosis after the occurrence of CAPS were excluded from this analysis and (2) individuals with psychosis are less likely to participate volunteer-based studies. As a result, the effect of psychosis vulnerability on risk of CAPS is possibly higher than what is reported in this study.

## Conclusions

Our study builds on previous investigations assessing predictors of psychotic symptoms induced by cannabis [15, 75, 81, 82], and is the first to have systematically investigated cannabis-associated psychotic symptoms, leading to emergency medical treatment in a large sample of PWUC. We showed that such acute adverse reactions can occur among PWUC, and that some individuals are at a particular high risk (e.g., young users consuming potent forms of cannabis, those with mental health problems). Given the increasing legalization of cannabis for recreational purposes, more efforts to educate young people and public health initiatives about the consequences of cannabis use are therefore needed, to minimize harms and safeguard PWUC.

## Supplementary information

Supplement
