# Supplementary material for: Rates and correlates of cannabis-associated psychotic symptoms in over 230,000 people who use cannabis
Source: Transl Psychiatry. 2022 Sep 6;12:369. doi: 10.1038/s41398-022-02112-8 (PMC9448725; doi:10.1038/s41398-022-02112-8)
Supplement: Supplementary file 1 — Supplement [file 41398_2022_2112_MOESM1_ESM.pdf]

Supplementary Material

SINTRODUCTION 2

Table 1. Previous studies examining rates of cannabis-associated outcomes 2

SMETHODS 3

Sample 3

Measures 3

Analysis 7

SRESULTS 9

Sample characteristics 9

Correlates of cannabis-associated psychotic symptoms 9

SDISCUSSION 11

Rates of CAPS 11

Sample representativeness 11

sTable 2. Global Drug Survey measures 13

sTable 3. Sample characteristics of included people who use cannabis 14

sTable 4. Rates of cannabis-associated psychotic symptoms (CAPS) requiring emergency medical treatment 15

sTable 5. Sensitivity analysis of rates of cannabis-associated psychotic symptoms (CAPS) requiring emergency medical treatment in individuals indicating to have not previously participated in the Global Drug Survey 16

sTable 6. Subgroup analyses assessing correlates of cannabis-associated psychotic symptoms (CAPS) 17

sTable 7. Sensitivity analysis of rates of cannabis-associated psychotic symptoms (CAPS) in subsets of people who use cannabis 19

sTable 8. Reasons for not seeking emergency medical treatment following cannabis use in individuals from the United States 20

sTable 9. Characterization of people who use cannabis and cannabis-associated psychotic symptoms (CAPS) 20

sTable 10. Subgroup analysis: Percentage of individuals requiring hospital admission as a result of cannabis-associated psychotic symptoms (CAPS) 21

SREFERENCES 21

sIntroduction

Table 1. Previous studies examining rates of cannabis-associated outcomes

Study	Country	Sample	Definition of cannabis-associated outcome	Rates
Jouanjus et al. (2010)[1]	France	200 patients with a diagnosis of mental and behavioural disorders due to use of cannabinoids	Annual incidence of cannabis-related hospitalizations	1.9 per 1000 PWUC (recent use)<br>3.2 per 1000 PWUC (regular use)
			Annual incidence of cannabis-related hospitalizations for psychiatric disorders	2.94 per 1000 PWUC
Schmid et al. (2019)[2]	Switzerland	700 individuals presenting to emergency departments	Emergency medical treatment due to acute toxicity related to cannabis use	0.1% of all cases presenting to emergency departments were related to acute toxicity of cannabis
Winstock et al. (2015)[3]	International	Survey assessing 21,200 individuals reporting cannabis use in the last year (including synthetic cannabis)	Seeking of emergency medical treatment following cannabis use	0.2% among PWUC sought emergency medical treatment in the previous year<br>1% of people using synthetic cannabis sought emergency medical treatment in the previous year
Arendt et al. (2005)[4]	Denmark	803 patients with a diagnosis of cannabis-induced psychotic disorder	Diagnosis of cannabis-induced psychotic disorder	2.7 incidence per 100 000 person-years (in the general population)
Hjorthøj et al. (2019)[5]	Denmark	4402 incident cases of cannabis-induced psychosis	Diagnosis of cannabis-induced psychotic disorder	incidence between 2.8 (2006) and 6.9 per 100 000 person-years (2014) (in the general population)

Note. PWUC = people who use cannabis

sMethods

Sample

Data was drawn from the largest online drug survey word-wide, the Global Drug Survey (GDS)[6], which collects annual cross-sectional data on drug use though an anonymous online survey. In our study, we included data from five years of GDS data collection (2014, 2015, 2016, 2017, 2019). 2018 data was not included as data on emergency medical treatment (EMT) was not collected that year. GDS uses an encrypted online platform to recruit its non-probability sample with the support of global media and harm reduction organisations. Further details on methods and limitations can be found in Barratt et al. (2017)[7]. Out of the total number of participants taking part in the five GDS years (N=529,574), we selected n=233,475 individuals, including those (1) with a history cannabis use, (2) who resided in a participating country that had a response rate of at least n=1000 PWUC and (3) had complete data on CAPS. Ethical approval was received from The Psychiatry, Nursing and Midwives Ethics subcommittee at Kings College, London (141/02), The University of Queensland (No: 2017001452) and The University of New South Wales (HREC HC17769).

Measures

Participants provided details on basic demographics (e.g., country of residence, gender, age, educational attainment, height and weight), self-reported mental health history and current treatment and an extensive battery of questions screening for substance and cannabis use (e.g. classes of substances used, frequency of use, preferred cannabis type). An overview of all included variables is provided below in sTable 2. The main outcome of our study –cannabis-associated psychotic-symptoms (CAPS) – was assessed using two items screening for cannabis-related emergency medical treatment, experienced either in the lifetime - “Have you ever sought emergency medical treatment following your use of cannabis?” or in the last year - “In the past 12 month, have you sought emergency medical treatment following your use of cannabis?”. Those individuals reporting any cannabis-related emergency medical treatment were asked to endorse from a list of 19 physical and psychological symptoms their symptom presentation: ”Which of the following did you present with:

aggression, anxiety, paranoia, and so forth", cf. below for a complete list of symptoms, or previous GDS publications analyzing data on emergency treatment[3,8]). Individuals who reported to have sought emergency medical treatment due to the occurrence of psychotic symptoms following cannabis use, including hallucinations ("seeing, hearing things") and/or paranoia ("paranoia, suspiciousness") were then classified as PWUC with CAPS. We used these two criteria, since a diagnosis of cannabis-induced psychotic disorder is given when hallucinations and/or delusions develop during cannabis intoxication[9]. Furthermore, the two items have been identified as the most reliable self-report measures screening for psychosis when validated against clinical interview measures[10]. To assess correlates of CAPS, we used data from a number of questions assessing factors that could plausibly link to cannabis-psychosis (e.g., age, mental health history, type of cannabis used). Finally, we analyzed data from items characterizing the CAPS event and its consequences (e.g., type and amount of cannabis used before seeking to emergency medical treatment for CAPS, time to recover, changes in cannabis use following the event).

Emergency medical treatment due to cannabis-associated psychotic symptoms (CAPS) was assessed using two items screening for cannabis-associated emergency medical treatment, experienced either in the last year ("In the past 12 month, have you sought emergency medical treatment following your use of cannabis?) or per lifetime ("Have you ever sought emergency medical treatment following your use of cannabis?"). Those individuals reporting cannabis-associated emergency medical treatment were asked to endorse from a list of 19 physical and psychological symptoms their symptom presentation (cf. box below). Individuals who reported to have sought emergency medical treatment due to the occurrence of psychotic symptoms following cannabis use, including hallucinations ("Seeing, hearing things") and paranoia ("Paranoia, suspiciousness") were then classified as people who use cannabis (PWUC) with CAPS (cf. box below, highlighted in bold).

BOX 1

"Thinking about the last time you sought emergency treatment following the use of cannabis, which of the following did you present with"

- Accident / trauma
- Extreme agitation
- Chest pain
- Extreme sweating
- **Seeing / hearing things**
- Thoughts or acts of self-harm
- Confusion
- Aggression / violence
- Palpitations
- Fits / seizures
- **Paranoia / suspiciousness**
- Headache
- Memory loss
- Anxiety / panic
- Nausea / vomiting
- Bladder / kidney problems
- Difficulty breathing
- Passed out / unconscious
- Very low mood in days afterwards

<u>Type of preferred cannabis</u> was assessed by asking participants about their preferred cannabis type ("Over the last 12 months which type of cannabis have used most commonly?"). Here, participants could select from a number of photos depicting different cannabis preparations. This data was used to classify participants into users of four types of cannabis[11], including (1) high-potency cannabis (e.g. skunk, use of the unpollinated flower), (2) herbal / normal weed (use of the pollinated flowers), (3) has/resin (compressed cannabis trichomes along with plant matter), (4) cannabis oil group (e.g. hash oil, butane hash oil). Using such classification system to infer data on the strength of cannabis has previously been validated[12,13].

<u>Route of administration (ROA) of cannabis:</u> ROA was assessed by asking "Which is the most common way you currently use cannabis?" and by providing a number of

selections from which participants could select their preferred route (e.g. smoked in a joint with tobacco, smoked in a joint without tobacco, smoked in blunt with tobacco, smoked in blunt without tobacco, smoked in a pipe with tobacco, smoked in a pipe without tobacco, smoked in a bong/water pipe with tobacco, smoked in a bong/water pipe without tobacco, smoked using 'bucket bong', smoked using hot knife, smoked using a vaporiser, ate it in food, drank it in tea/infusion). First, this data was used to classify users according to five routes of administration (joint/blunt, vape, pipe/bong, eat, drink, knife). Second, this data was used to categorize PWUC into those mixing cannabis with tobacco and those not mixing cannabis with tobacco.

Frequency of cannabis use in the last year: Participants were asked about the number of days they had used cannabis in the previous year ("How many days have you used cannabis in the last 12 months?"). Individuals were classified as high frequency PWUC if they reported to have used cannabis for more than 100 days in the last year.

Other drug use in the last year: Frequency of alcohol use was assessed using the question "In the last 12 months, how often do you have a drink containing alcohol?". Participants were classified as 'high frequency alcohol users" if they used alcohol 4 or more times per week. In addition, participants were classified into groups based on whether they had used any of the following substances in the past year: Cocaine, MDMA, amphetamine, methamphetamine, ketamine or LSD.

BMI: Body Mass Index (BMI) was defined as an individual's weight (kg), divided by the square of the individual's height (m^2). Individuals were categorized as underweight (BMI < 18.5), normal weight (BMI $\geq$ 18.5 and < 25) or overweight (BMI $\geq$ 25) , using the BMI classification for adults as provided by the Word Health Organization[14]. The interquartile range method was used to identify outliers.

Mental health diagnosis: Participants were asked about previous mental health diagnoses ("Have you ever been diagnosed with a mental illness?"). Those who reported to have a history of mental illness were then asked to indicate the diagnosis they received ("Which illnesses have you ever been diagnosed with? ADHD, depression, anxiety, bipolar, psychosis"). New dichotomized variables were created

for each of the mental health diagnoses, classifying individuals as cases with the disorder of interest (e.g., ADHD) and individuals without any history of mental illness. For diagnosis of psychosis, we excluded individuals with prolonged psychotic symptoms (longer than 4 weeks) following emergency medical treatment due the occurrence of CAPS. This was done to ensure that individuals who developed psychosis as a result of CAPS were not included in analyses assessing predictors of CAPS.

Use of cannabis for medicinal reasons: Participants were asked about their reasons for using cannabis and we classified individuals based on whether they indicated to use cannabis either for mostly medical reasons ("I use cannabis most of the time for medical reasons and sometimes for recreational purposes", "I use cannabis exclusively for medical reasons") or mostly recreational reasons ("I use cannabis exclusively for recreational purposes", "I use cannabis sometimes for medical reasons and most of the time for recreational purposes").

Exercise: Past year exercise was assessed by asking "How often in the last year did you exercise?", which was used to create a binary variable indicating either regular exercise ("once or twice a week", "more than 4 times a week") or little exercise ("never", "less than once every 3 months", "once every 3 months", "once a month")

Educational attainment: Educational attainment was assessed as a categorical variable, asking individuals to indicate their highest academic qualification attained. We classified individuals according to whether they completed higher education (at least bachelor degree, including those that reported to currently study) and those not having attained higher education. Of note, educational attainment in GDS 2014 was assessed in a different format compared to the other years of GDS and was therefore not included in our subset analysis exploring correlates of rates of CAPS.

Analysis

To assess if rates of CAPS differed across subgroups of PWUC, we grouped PWUC according to a number of factors that could plausibly link to risk of CAPS. For dichotomised variables (e.g. gender), we estimated the rates of CAPS in one

category (e.g. female gender) and compared them to the rates estimated for the corresponding reference group (e.g. male gender). To quantify the magnitude of differences in rates of CAPS between subgroups, we estimated risk ratios and the corresponding 95% CI and *p*-values as implemented in the R package fmsb[15].

For non-binary categorical variables (e.g. preferred type of cannabis used), the reference groups were defined as follows:

- For preferred type of cannabis (categories: high-potency cannabis, normal herbal cannabis, hash/resin, edible, oil), we used ‘normal herbal cannabis’ as the reference group and compared the rates of CAPS in individuals using normal herbal cannabis to rates estimated for individuals using other cannabis types separately, namely 1) high potency versus normal herbal cannabis, 2) hash/resin versus normal herbal cannabis, 3) edible versus normal herbal cannabis and 4) cannabis oil versus normal herbal cannabis.
- For country of residence, we selected subgroups according to a specific country (e.g. individuals residing in Germany) and used the remaining sample as the reference group (i.e. individuals not residing Germany).
- For route of administration (ROA) of cannabis use, we grouped together PWUC that predominantly used smoked cannabis in a joint/blunt as the reference group and compared them to the other ROAs assessed in this survey (e.g. vape, food, cf. above)

A complete list of the assessed subgroups, as well as their respective reference group is provided in sTable 6 (below).

sResults

Sample characteristics

233,475 participants with indicated cannabis use and data on CAPS were included in this study. Sample characteristics are displayed in sTable 3. The majority of the sample was male (72%) and 25 years of age or younger (58%). Germany was the country showing the largest response rate (26%), followed by the United States (11%) and the United Kingdom (8%). The most commonly used type of cannabis in this sample was herbal cannabis (47%), with joint/blunt being the preferred route of administration (75%). A substantial proportion (43%) of PWUC reported frequent use of cannabis in the last year (more than 100 days of cannabis use).

Correlates of cannabis-associated psychotic symptoms

Rates of CAPS were also higher in PWUC residing in Denmark (RR=3.01), when compared to the rest of the sample. To assess potential explanations for the differences in rates of CAPS across the two countries (Denmark and the US), we conducted a number of exploratory analyses (cf. below, sTable 7-8). The results indicated that in Denmark, the elevated rates of CAPS may reflect the popularity of high-potency resin in this country (e.g. 58% of Danish PWUC reported the use of mostly resin/hash), which typically contains 23% THC or more since 2014[16]. In line with this, rates of CAPS dropped when excluding PWUC from Denmark that reported to use resin (i.e. 0.66% risk of CAPS in Danish PWUC using resin vs. 0.21% risk of CAPS in non-Danish PWUC using resin, cf. sTable 7). As such, there is considerable evidence for the notion that the use of high-potency Danish resin links to risk of CAPS.

Lower rates of CAPS were present in PWUC residing in the United States (RR=0.4), which could be due to a number of reasons, including the higher costs of care that may prevent people from seeking emergency medical treatment in US, or differences in the preparation of cannabis between the US and the other countries included in this study. To assess if healthcare cost explained the reduced rates in the US, we analysed data from US individuals on reasons for not seeking emergency medical treatment after the occurrence of CAPS. The results highlight that PWUC typically

did not seek emergency medical treatment because they knew they would get better (36%), they were taken care of by friends (35%) or they thought their condition was not serious enough (26%) (cf. sTable 8 for all estimates).

sDiscussion

Rates of CAPS

Comparing rates of CAPS in a number of countries, we found that rates were lower in PWUC residing in the United States, which could be due to a number of reasons. First, differences in the potency of cannabis are unlikely to contribute to variations in rates of CAPS, considering that herbal cannabis, the most popular type of cannabis used in the US, is of similar potency in the United States and Europe (17% THC in vs 14% THC in 2017[17], respectively). Although novel high-potency forms of cannabis gain increasing popularity in the US (e.g. butane hash oil that is typically linked to stronger adverse experiences[18]), the number of individuals using such types is still comparatively low. Instead, the lower rates could mirror the financial barriers of seeking emergency medical treatment in the US, where treatment can be associated with substantial costs[19]. To assess if healthcare cost explained the reduced rates of CAPS in the United States, we analysed data from individuals residing in the US who provided reasons for not seeking emergency medical treatment. The results show that most commonly individuals did not seek emergency medical treatment because they knew they would get better (36%), they were taken care of by friends (35%) or thought their condition was not serious enough (26%) (cf. sTable 8 for all estimates). However, one main difference between PWUC from Europe and the US lies in their preparation of cannabis: in Europe, cannabis is typically mixed with tobacco, while users from the US consume cannabis without tobacco[20,21]. As such, cannabis combined with tobacco may carry a greater risk for CAPS when compared to cannabis alone.

Sample representativeness

Finally, data was collected using non-representative sampling, which has advantages and disadvantages including those surrounding reliability and validity at a population-based level, as discussed elsewhere[22–24]. However, the anonymous web survey design employed here is particularly valuable for the collection of data on sensitive topics including substance use, as such design can provide a sense of privacy that is missing in face-to-face interviews. In addition, our study was set out to analyze data from a large sample restricted to people who report recreational cannabis use. This selected sample has been shown to have similar characteristics

to samples of cannabis users produced by probability-based sampling[7]. While cannabis use appears more prevalent in the GDS compared to household surveys (70% vs. 34% reporting ever cannabis use, respectively), differences are likely due to the younger population sampled by the GDS. When stratifying according to age, the demographic distribution and cannabis use prevalence among PWUC taking part in the GDS compares to those taking part in probability-based sampling surveys[7].

sTable 2. Global Drug Survey measures

Variable	Questionnaire item	2014	2015	2016	2017	2019
Country	Which country do you currently live in?	Y	Y	Y	Y	Y
Age	How old are you?	Y	Y	Y	Y	Y
Gender	Are you male / female / transgender?	Y	Y	Y	Y	Y
Ethnicity	What is your ethnicity?	Y	Y	Y	Y	Y
Educational attainment	What is your highest academic qualification attained?	N	Y	Y	Y	Y
Studying	Are you currently studying	Y	Y	Y	Y	Y
Height	What is your height in cm?	Y	Y	Y	Y	Y
Weight	What is your weight in kg?	Y	Y	Y	Y	Y
Exercise	How often in the last year did you exercise?	Y	Y	Y	Y	Y
Cannabis use (ever)	Have you ever used cannabis?	Y	Y	Y	Y	Y
Cannabis frequency	How many days have you used cannabis in the last 12 months?	Y	Y	Y	Y	Y
Cannabis type	Over the last 12 months which type of cannabis have used most commonly?	Y	Y	Y	Y	Y
Medical cannabis use	What are your reasons for using cannabis?	Y	Y	Y	Y	Y
Route of cannabis use	Which is the most common way you currently use cannabis? [e.g. smoke in bong, smoke in joint etc]	Y	Y	Y	Y	N
Route of cannabis use - mixed with tobacco	Do you add tobacco to your cannabis when preparing it yourself?	Y	Y	Y	Y	Y
Alcohol frequency	In the last 12 months, how often do you have a drink containing alcohol?	Y	Y	Y	Y	Y
Cocaine use	Did you use cocaine in the last year?	Y	Y	Y	Y	Y
MDMA use	Did you use MDMA in the last year?	Y	Y	Y	Y	Y
Amphetamine use	Did you use amphetamines in the last year?	Y	Y	Y	Y	Y
Methamphetamine use	Did you use methamphetamine in the last year?	Y	Y	Y	Y	Y
Ketamine use	Did you use ketamine in the last year?	Y	Y	Y	Y	Y
LSD use	Did you use LSD in the last year?	Y	Y	Y	Y	Y
Lifetime cannabis-related emergency medical treatment	Have you ever sought emergency medical help after using cannabis?	N	Y	Y	N	N
Last-year cannabis-related emergency medical treatment	In the last 12 months have you sought emergency treatment following the use of cannabis?	Y	N	N	Y	Y
Cannabis-associated psychotic symptoms (hallucinations)	When seeking emergency medical treatment following cannabis, did you present with hallucinations?	Y	Y	Y	Y	Y
Cannabis-associated psychotic symptoms (paranoia)	When seeking emergency medical treatment following cannabis, did you present with paranoia/suspiciousness?	Y	Y	Y	Y	Y
Hospital admission following emergency medical treatment for cannabis-associated psychotic symptoms	Were you admitted to a hospital after seeking emergency medical treatment following cannabis use?	Y	Y	Y	Y	Y
Reductions in cannabis use following emergency medical treatment for cannabis-associated psychotic symptoms	How has the experience impacted on your use of cannabis - did you cut down on your use of cannabis?	Y	Y	Y	Y	Y
Other drug use before seeking emergency medical treatment for cannabis-associated psychotic symptoms	Which other drugs have you taken before seeking emergency medical treatment following cannabis use?	Y	Y	Y	Y	N
Amount of cannabis before seeking emergency medical treatment for cannabis-associated psychotic symptoms	How much cannabis have you used before seeking emergency medical treatment following cannabis use? (reported in mg)	Y	N	N	Y	Y
Type of cannabis used before seeking emergency medical treatment for cannabis-associated psychotic symptoms	What type of cannabis have you used before seeking emergency medical treatment?	N	Y	Y	Y	Y
Amount of alcohol before seeking emergency medical treatment for cannabis-associated psychotic symptoms	How many alcoholic drinks did you drink that night?	N	N	N	Y	Y
Time to recovery	How long before you felt back to normal following cannabis-associated psychotic symptoms	Y	Y	Y	Y	Y
Mental health history (depression)	Have you ever been diagnosed with depression?	Y	Y	Y	Y	Y
Mental health history (anxiety)	Have you ever been diagnosed with anxiety?	Y	Y	Y	Y	Y
Mental health history (bipolar disorder)	Have you ever been diagnosed with bipolar disorder?	Y	Y	Y	Y	Y
Mental health history (psychosis)	Have you ever been diagnosed with psychosis?	Y	Y	Y	Y	Y
Mental health history (ADHD)	Have you ever been diagnosed with ADHD?	Y	Y	Y	Y	Y

Note. Availability of measurements across the different years of GDS data collection. Y = Item available, N = Item not available.

sTable 3. Sample characteristics of included people who use cannabis

Group	Sample characteristic	Number of PWUC [all]	Percentage (%) [all]	Number of PWUC [last year CAPS]	Percentage (%) [last year CAPS]	Number of PWUC [lifetime CAPS]	Percentage (%) [lifetime CAPS]
		233475	100	148109	100	85366	100
Year	2014	33378	14.3	33378	22.54		
	2015	39938	17.11			39938	46.78
	2016	45428	19.46			45428	53.22
	2017	57001	24.41	57001	38.49		
	2019	57730	24.73	57730	38.98		
Gender*	Female	63441	27.53	39559	26.99	23882	28.46
	Male	167037	72.47	107002	73.01	60035	71.54
Age	16 or younger	6980	3.01	4638	3.14	2342	2.77
	21 or younger	76108	32.79	47016	31.85	29092	34.42
	25 or younger	51864	22.34	31721	21.49	20143	23.84
	30 or younger	38307	16.5	24760	16.77	13547	16.03
	40 or younger	35084	15.11	23500	15.92	11584	13.71
	50 or younger	13865	5.97	9283	6.29	4582	5.42
	Older than 50	9926	4.28	6707	4.54	3219	3.81
Country	Australia	12006	5.14	7870	5.31	4136	4.85
	Austria	6490	2.78	4670	3.15	1820	2.13
	Belgium	3210	1.37	1912	1.29	1298	1.52
	Brazil	7119	3.05	3696	2.5	3423	4.01
	Canada	7307	3.13	5522	3.73	1785	2.09
	Colombia	3129	1.34	1497	1.01	1632	1.91
	Denmark	10226	4.38	9764	6.59	462	0.54
	Finland	2225	0.95	1958	1.32	267	0.31
	France	12325	5.28	4658	3.14	7667	8.98
	Germany	61135	26.18	39388	26.59	21747	25.48
	Greece	1232	0.53	1097	0.74	135	0.16
	Hungary	6533	2.8	3556	2.4	2977	3.49
	Ireland	3558	1.52	2155	1.46	1403	1.64
	Italy	7670	3.29	5812	3.92	1858	2.18
	Mexico	2691	1.15	1657	1.12	1034	1.21
	Netherlands	8837	3.78	3801	2.57	5036	5.9
	New Zealand	10144	4.34	6641	4.48	3503	4.1
	Norway	2134	0.91	1033	0.7	1101	1.29
	Portugal	1860	0.8	1038	0.7	822	0.96
	Slovakia	1167	0.5	1108	0.75	59	0.07
	Spain	3292	1.41	1375	0.93	1917	2.25
	Sweden	1771	0.76	929	0.63	842	0.99
	Switzerland	11336	4.86	6750	4.56	4586	5.37
	United Kingdom	19561	8.38	11859	8.01	7702	9.02
	United States	26517	11.36	18363	12.4	8154	9.55
Preferred type of cannabis used (last 12 months)	herbal cannabis	103705	46.94	60289	43.42	43416	52.89
	high potency cannabis	90421	40.93	60271	43.41	30150	36.73
	resin/hash	23355	10.57	15398	11.09	7957	9.69
	oil group	3455	1.56	2888	2.08	567	0.69
	drink	319	0.18	169	0.19	150	0.18

Most common route of administration (ROA	eaten	2660	1.54	1458	1.64	1202	1.44
	joint/blunt	129024	74.68	65045	72.99	63979	76.49
	knife	349	0.2	171	0.19	178	0.21
	pipe/bong	32452	18.78	17721	19.88	14731	17.61
	vape	7963	4.61	4556	5.11	3407	4.07
Number of days of cannabis use (last 12 months)	10 days or less	55306	23.9	36158	24.41	19148	22.99
	11-50 days	48373	20.9	30376	20.51	17997	21.6
	51-100 days	28883	12.48	16673	11.26	12210	14.66
	101 days or more	98852	42.72	64902	43.82	33950	40.75

Note. Out of the total number of participants (N=529,574) taking part in the five GDS surveys (2014, 2015, 2016, 2017, 2019), we only included participants (1) with a history cannabis use, (2) who resided in a participating country with a response rate of at least n=1000 and (3) had complete data on cannabis-associated psychotic symptoms (CAPS). The final dataset comprised people who use cannabis (PWUC) with data on CAPS requiring emergency medical treatment, including PWUC with data on last-year (n=148,109) or lifetime (n=85,366) occurrence of CAPS. Displayed in the table are the demographic information of the sample as a whole (cf. columns labelled as [all]), as well as demographic information separated out for the sub-sample including only PWUC with data on last year CAPS (cf. columns labelled as [last year CAPS]) and the sub-sample including only PWUC with data on lifetime CAPS (cf. columns labelled as [lifetime CAPS]).

* Not reported are frequency estimates for individuals self-identifying as non-binary gender as this data was not assessed in each year of data collection.

sTable 4. Rates of cannabis-associated psychotic symptoms (CAPS) requiring emergency medical treatment

	**Reason for seeking emergency medical treatment**	**% (95% CI)**	**PWUC with CAPS**	**Total sample of PWUC**
Last year occurrence	Any adverse event	0.59 (0.55; 0.63)	868	148109
Lifetime occurrence	Any adverse event	1.11 (1.04; 1.19)	951	85366
Last year occurrence	CAPS - hallucinations and paranoia	0.07 (0.06; 0.08)	101	148109
Lifetime occurrence	CAPS - hallucinations and paranoia	0.15 (0.12; 0.18)	127	84814
Last year occurrence	CAPS - hallucinations only	0.02 (0.01; 0.03)	29	148109
Lifetime occurrence	CAPS - hallucinations only	0.07 (0.05; 0.09)	56	84814
Last year occurrence	CAPS - paranoia only	0.10 (0.09; 0.12)	150	148109
Lifetime occurrence	CAPS - paranoia only	0.25 (0.22; 0.29)	216	84814
Last year occurrence	CAPS - psychotic symptoms	0.19 (0.17; 0.21)	280	148109
Lifetime occurrence	CAPS - psychotic symptoms	0.47 (0.42; 0.52)	399	85366

Note. Shown are the rates people who use cannabis (PWUC) requiring emergency medical treatment following the use of cannabis, estimated for different symptom profiles. 'Any adverse event' includes all participants reporting to have sought emergency medical treatment following the use of cannabis in the last year or in their lifetime. 'CAPS - psychotic symptoms' includes PWUC reporting CAPS, defined as the occurrence of hallucinations or paranoia requiring emergency medical treatment. Two datasets were analysed separately, including 1) a sample of PWUC with data on last year occurrence of CAPS (n=148,109) and 2) and a sample of PWUC with data on lifetime occurrence of CAPS (n=85,366).

sTable 5. Sensitivity analysis of rates of cannabis-associated psychotic symptoms (CAPS) requiring emergency medical treatment in individuals indicating to have not previously participated in the Global Drug Survey

**Sample**	**Reason for seeking emergency medical treatment**	**% (95% CI)**	**n PWUCs with CAPS**	**n sample**
Last year occurrence	Any adverse events	0.53 (0.49; 0.58)	507	95159
Lifetime occurrence	Any adverse events	1.12 (1.04; 1.20)	816	72892
Last year occurrence	CAPS - hallucinations and paranoia	0.07 (0.06; 0.09)	68	95159
Lifetime occurrence	CAPS - hallucinations and paranoia	0.15 (0.13; 0.18)	111	72432
Last year occurrence	CAPS - hallucinations only	0.02 (0.01; 0.03)	16	95159
Lifetime occurrence	CAPS - hallucinations only	0.07 (0.06; 0.10)	54	72432
Last year occurrence	CAPS - paranoia only	0.11 (0.09; 0.13)	106	95159
Lifetime occurrence	CAPS - paranoia only	0.26 (0.23; 0.30)	191	72432
Last year occurrence	CAPS - psychotic symptoms	0.20 (0.17; 0.23)	190	95159
Lifetime occurrence	CAPS - psychotic symptoms	0.49 (0.44; 0.54)	356	72892

Note. Sensitivity analysis excluding individuals who indicated to have participated in previous years of the Global Drug Survey (GDS). More specifically, we included all people who use cannabis (PWUC) taking part in GDS 2014 and excluded PWUC taking part in any of the subsequent GDS years (i.e. 2015, 2016, 2017, 2019) if previous GDS participation was indicated. Of the whole sample initially included in this study (n=233,475, cf. sTable 3), we restricted the sample to n=168,051 (71.98%) who indicated not to have taken part in any previous GDS.

sTable 6. Subgroup analyses assessing correlates of cannabis-associated psychotic symptoms (CAPS)

Category	Sub-groups	% (95% CI) reporting CAPS	n PWUCs with CAPS	n sample	RR (95% CI)	*p*-value (RR)	*p*-value (RR, fdr corrected)	Comparison
gender	male (comparison group)	0.17 (0.14; 0.19)	177	107002	0.69 (0.54; 0.88)	0.003	0.177	male versus female
	female (reference group)	0.24 (0.19; 0.29)	95	39559				
age	21 years of age or younger (comparison group)	0.32 (0.27; 0.37)	163	51654	2.66 (2.09; 3.37)	<0.001	<0.001	21 years of age or younger versus older than 21 years of age
	older than 21 years of age (reference group)	0.12 (0.10; 0.14)	114	95971				
level of exercise	little exercise (comparison group)	0.19 (0.16; 0.23)	107	55773	1.18 (0.90; 1.53)	0.225	1.000	little exercise versus regular exercise
	regular exercise (reference group)	0.16 (0.13; 0.20)	114	69959				
education	no higher education (comparison group)	0.13 (0.08; 0.18)	28	22197	0.74 (0.48; 1.14)	0.173	1.000	no higher education versus higher education
	higher education (reference group)	0.17 (0.14; 0.21)	84	49460				
BMI	underweight (comparison group)	0.21 (0.14; 0.29)	31	14962	1.08 (0.72; 1.62)	0.724	1.000	underweight versus normal weight
	normal weight (reference group)	0.19 (0.16; 0.24)	92	47773				
BMI	overweight (comparison group)	0.18 (0.14; 0.23)	65	36668	0.92 (0.67; 1.26)	0.609	1.000	overweight versus normal weight
	normal weight (reference group)	0.19 (0.16; 0.24)	92	47773				
country	PWUC from Australia (comparison group)	0.18 (0.10; 0.30)	14	7870	0.94 (0.55; 1.60)	0.815	1.000	PWUC from Australia versus PWUC not from Australia
	PWUC not from Australia (reference group)	0.19 (0.17; 0.21)	266	140239				
country	PWUC from Austria (comparison group)	0.21 (0.10; 0.39)	10	4670	1.14 (0.61; 2.14)	0.689	1.000	PWUC from Austria versus PWUC not from Austria
	PWUC not from Austria (reference group)	0.19 (0.17; 0.21)	270	143439				
country	PWUC from Belgium (comparison group)	0.21 (0.06; 0.53)	4	1912	1.11 (0.41; 2.97)	0.838	1.000	PWUC from Belgium versus PWUC not from Belgium
	PWUC not from Belgium (reference group)	0.19 (0.17; 0.21)	276	146197				
country	PWUC from Brazil (comparison group)	0.30 (0.15; 0.53)	11	3696	1.60 (0.88; 2.92)	0.127	1.000	PWUC from Brazil versus PWUC not from Brazil
	PWUC not from Brazil (reference group)	0.19 (0.16; 0.21)	269	144413				
country	PWUC from Canada (comparison group)	0.27 (0.15; 0.45)	15	5522	1.46 (0.87; 2.46)	0.152	1.000	PWUC from Canada versus PWUC not from Canada
	PWUC not from Canada (reference group)	0.19 (0.16; 0.21)	265	142587				
country	PWUC from Colombia (comparison group)	0.40 (0.15; 0.87)	6	1497	2.14 (0.96; 4.81)	0.064	1.000	PWUC from Colombia versus PWUC not from Colombia
	PWUC not from Colombia (reference group)	0.19 (0.17; 0.21)	274	146612				
country	PWUC from Denmark (comparison group)	0.50 (0.37; 0.66)	49	9764	3.01 (2.21; 4.09)	<0.001	<0.001	PWUC from Denmark versus PWUC not from Denmark
	PWUC not from Denmark (reference group)	0.17 (0.15; 0.19)	231	138345				
country	PWUC from Finland (comparison group)	0.05 (0.00; 0.28)	1	1958	0.27 (0.04; 1.90)	0.188	1.000	PWUC from Finland versus PWUC not from Finland
	PWUC not from Finland (reference group)	0.19 (0.17; 0.21)	279	146151				
country	PWUC from France (comparison group)	0.09 (0.02; 0.22)	4	4658	0.45 (0.17; 1.20)	0.109	1.000	PWUC from France versus PWUC not from France
	PWUC not from France (reference group)	0.19 (0.17; 0.22)	276	143451				
country	PWUC from Germany (comparison group)	0.24 (0.19; 0.29)	93	39388	1.37 (1.07; 1.76)	0.012	0.659	PWUC from Germany versus PWUC not from Germany
	PWUC not from Germany (reference group)	0.17 (0.15; 0.20)	187	108721				
country	PWUC from Greece (comparison group)	0.27 (0.06; 0.80)	3	1097	1.45 (0.47; 4.52)	0.520	1.000	PWUC from Greece versus PWUC not from Greece
	PWUC not from Greece (reference group)	0.19 (0.17; 0.21)	277	147012				
country	PWUC from Hungary (comparison group)	0.06 (0.01; 0.20)	2	3556	0.29 (0.07; 1.17)	0.083	1.000	PWUC from Hungary versus PWUC not from Hungary
	PWUC not from Hungary (reference group)	0.19 (0.17; 0.22)	278	144553				
country	PWUC from Ireland (comparison group)	0.09 (0.01; 0.33)	2	2155	0.49 (0.12; 1.96)	0.311	1.000	PWUC from Ireland versus PWUC not from Ireland
	PWUC not from Ireland (reference group)	0.19 (0.17; 0.21)	278	145954				
country	PWUC from Italy (comparison group)	0.05 (0.01; 0.15)	3	5812	0.27 (0.09; 0.83)	0.022	1.000	PWUC from Italy versus PWUC not from Italy

	PWUC not from Italy (reference group)	0.19 (0.17; 0.22)	277	142297				
country	PWUC from Mexico (comparison group)	0.12 (0.01; 0.44)	2	1657	0.64 (0.16; 2.55)	0.523	1.000	PWUC from Mexico versus PWUC not from Mexico
	PWUC not from Mexico (reference group)	0.19 (0.17; 0.21)	278	146452				
country	PWUC from Netherlands (comparison group)	0.16 (0.06; 0.34)	6	3801	0.83 (0.37; 1.87)	0.654	1.000	PWUC from Netherlands versus PWUC not from Netherlands
	PWUC not from Netherlands (reference group)	0.19 (0.17; 0.21)	274	144308				
country	PWUC from New Zealand (comparison group)	0.03 (0.00; 0.11)	2	6641	0.15 (0.04; 0.62)	0.008	0.435	PWUC from New Zealand versus PWUC not from New Zealand
	PWUC not from New Zealand (reference group)	0.20 (0.17; 0.22)	278	141468				
country	PWUC from Norway (comparison group)	0.19 (0.02; 0.7)	2	1033	1.02 (0.26; 4.11)	0.973	1.000	PWUC from Norway versus PWUC not from Norway
	PWUC not from Norway (reference group)	0.19 (0.17; 0.21)	278	147076				
country	PWUC from Portugal (comparison group)	0.58 (0.21; 1.25)	6	1038	3.10 (1.38; 6.95)	0.006	0.314	PWUC from Portugal versus PWUC not from Portugal
	PWUC not from Portugal (reference group)	0.19 (0.16; 0.21)	274	147071				
country	PWUC from Slovakia (comparison group)	0.09 (0.00; 0.50)	1	1108	0.48 (0.07; 3.38)	0.458	1.000	PWUC from Slovakia versus PWUC not from Slovakia
	PWUC not from Slovakia (reference group)	0.19 (0.17; 0.21)	279	147001				
country	PWUC from Spain (comparison group)	0.29 (0.08; 0.74)	4	1375	1.55 (0.58; 4.14)	0.386	1.000	PWUC from Spain versus PWUC not from Spain
	PWUC not from Spain (reference group)	0.19 (0.17; 0.21)	276	146734				
country	PWUC from Sweden (comparison group)	0.11 (0.00; 0.60)	1	929	0.57 (0.08; 4.04)	0.572	1.000	PWUC from Sweden versus PWUC not from Sweden
	PWUC not from Sweden (reference group)	0.19 (0.17; 0.21)	279	147180				
country	PWUC from Switzerland (comparison group)	0.19 (0.10; 0.33)	13	6750	1.02 (0.58; 1.78)	0.945	1.000	PWUC from Switzerland versus PWUC not from Switzerland
	PWUC not from Switzerland (reference group)	0.19 (0.17; 0.21)	267	141359				
country	PWUC from United Kingdom (comparison group)	0.09 (0.05; 0.17)	11	11859	0.47 (0.26; 0.86)	0.014	0.743	PWUC from United Kingdom versus PWUC not from United Kingdom
	PWUC not from United Kingdom (reference group)	0.20 (0.17; 0.22)	269	136250				
country	PWUC from United States (comparison group)	0.08 (0.05; 0.13)	15	18363	0.40 (0.24; 0.67)	0.001	0.029	PWUC from United States versus PWUC not from United States
	PWUC not from United States (reference group)	0.20 (0.18; 0.23)	265	129746				
reason for use	cannabis used for recreational reasons (comparison group)	0.18 (0.16; 0.21)	252	136658	0.89 (0.57; 1.39)	0.608	1.000	cannabis used for recreational reasons versus cannabis used for medical reasons
	cannabis used for medical reasons (reference group)	0.21 (0.13; 0.32)	21	10137				
type of cannabis	high potency cannabis (comparison group)	0.17 (0.14; 0.21)	102	60271	0.96 (0.73; 1.26)	0.783	1.000	high potency cannabis versus herbal cannabis
	herbal cannabis (reference group)	0.18 (0.14; 0.21)	106	60289				
type of cannabis	resin/hash (comparison group)	0.37 (0.28; 0.48)	57	15398	2.11 (1.53; 2.90)	<0.001	0.000	resin/hash versus herbal cannabis
	herbal cannabis (reference group)	0.18 (0.14; 0.21)	106	60289				
type of cannabis	oil group (comparison group)	0.07 (0.01; 0.25)	2	2888	0.39 (0.10; 1.59)	0.192	1.000	oil group versus herbal cannabis
	herbal cannabis (reference group)	0.18 (0.14; 0.21)	106	60289				
cannabis frequency	high frequency cannabis use (comparison group)	0.17 (0.14; 0.21)	111	64902	0.84 (0.66; 1.07)	0.159	1.000	high frequency cannabis use versus low frequency cannabis use
	low frequency cannabis use (reference group)	0.20 (0.17; 0.24)	169	83207				
cannabis mix	cannabis mixed with tobacco (comparison group)	0.28 (0.24; 0.33)	163	58016	2.15 (1.68; 2.74)	<0.001	<0.001	cannabis mixed with tobacco versus cannabis not mixed with tobacco
	cannabis not mixed with tobacco (reference group)	0.13 (0.11; 0.16)	106	81066				
preferred ROA	eaten (comparison group)	0.21 (0.04; 0.60)	3	1458	0.83 (0.26; 2.59)	0.743	1.000	eaten versus joint/blunt
	joint/blunt (reference group)	0.25 (0.21; 0.29)	162	65045				
preferred ROA	knife (comparison group)	0.58 (0.01; 3.22)	1	171	2.35 (0.33; 16.67)	0.393	1.000	knife versus joint/blunt
	joint/blunt (reference group)	0.25 (0.21; 0.29)	162	65045				
preferred ROA	pipe/bong (comparison group)	0.21 (0.15; 0.29)	38	17721	0.86 (0.60; 1.23)	0.406	1.000	pipe/bong versus joint/blunt
	joint/blunt (reference group)	0.25 (0.21; 0.29)	162	65045				
preferred ROA	vape (comparison group)	0.07 (0.01; 0.19)	3	4556	0.26 (0.08; 0.83)	0.022	1.000	vape versus joint/blunt
	joint/blunt (reference group)	0.25 (0.21; 0.29)	162	65045				

other drug use	high frequency alcohol use (comparison group)	0.11 (0.07; 0.17)	22	19868	0.56 (0.36; 0.86)	0.008	0.440	high frequency alcohol use versus low frequency alcohol use
	low frequency alcohol use (reference group)	0.20 (0.18; 0.23)	252	126595				
other drug use	cocaine use (comparison group)	0.20 (0.16; 0.25)	96	47537	0.89 (0.67; 1.17)	0.396	1.000	cocaine use versus no cocaine use
	no cocaine use (reference group)	0.23 (0.19; 0.28)	97	42510				
other drug use	MDMA use (comparison group)	0.24 (0.21; 0.29)	145	59634	1.46 (1.08; 1.97)	0.014	0.745	MDMA use versus no MDMA use
	no MDMA use (reference group)	0.17 (0.13; 0.21)	60	35962				
other drug use	amphetamine use (comparison group)	0.27 (0.22; 0.33)	91	33905	1.29 (0.97; 1.73)	0.080	1.000	amphetamine use versus no amphetamine use
	no amphetamine use (reference group)	0.21 (0.17; 0.25)	93	44847				
other drug use	methamphetamine use (comparison group)	0.31 (0.17; 0.51)	15	4861	1.20 (0.70; 2.07)	0.502	1.000	methamphetamine use versus no methamphetamine use
	no methamphetamine use (reference group)	0.26 (0.21; 0.31)	99	38623				
other drug use	ketamine use (comparison group)	0.16 (0.11; 0.22)	31	19974	0.58 (0.39; 0.87)	0.008	0.412	ketamine use versus no ketamine use
	no ketamine use (reference group)	0.27 (0.22; 0.32)	106	39681				
other drug use	LSD use (comparison group)	0.19 (0.15; 0.24)	61	32176	0.79 (0.58; 1.08)	0.137	1.000	LSD use versus no LSD use
	no LSD use (reference group)	0.24 (0.20; 0.29)	115	47951				
mental health diagnosis	diagnosis of depression (comparison group)	0.32 (0.25; 0.41)	71	21913	2.68 (2.00; 3.61)	<0.001	<0.001	diagnosis of depression versus no mental health diagnosis
	no mental health diagnosis (reference group)	0.12 (0.1; 0.14)	115	95253				
mental health diagnosis	diagnosis of anxiety (comparison group)	0.35 (0.27; 0.46)	55	15592	2.92 (2.12; 4.03)	<0.001	<0.001	diagnosis of anxiety versus no mental health diagnosis
	no mental health diagnosis (reference group)	0.12 (0.1; 0.14)	115	95253				
mental health diagnosis	diagnosis of bipolar (comparison group)	0.52 (0.3; 0.84)	16	3085	4.30 (2.55; 7.24)	<0.001	<0.001	diagnosis of bipolar versus no mental health diagnosis
	no mental health diagnosis (reference group)	0.12 (0.1; 0.14)	115	95253				
mental health diagnosis	diagnosis of psychosis** (comparison group)	1.69 (1.09; 2.51)	24	1419	14.01 (9.05; 21.68)	<0.001	<0.001	diagnosis of psychosis** versus no mental health diagnosis
	no mental health diagnosis (reference group)	0.12 (0.1; 0.14)	115	95253				
mental health diagnosis	diagnosis of ADHD (comparison group)	0.26 (0.14; 0.44)	14	5321	2.18 (1.25; 3.79)	0.006	0.311	diagnosis of ADHD versus no mental health diagnosis
	no mental health diagnosis (reference group)	0.12 (0.1; 0.14)	115	95253				

Note. The sample was restricted to people who use cannabis (PWUC) with data on last year cannabis-associated psychotic symptoms (CAPS) (n = 148,109). The reported *p*-values (cf. column '*p*-value RR') corresponds to the *p*-value of the Risk Ratios (RR). '*p*-value (RR, fdr corrected)' indexes the FDR (False Discovery Rate) corrected *p*-values estimates adjusted for multiple testing (k=53 comparisons). ROA = Route of administration.

** Subgroup of PWUC who reported to have been diagnosed with psychosis excludes those individuals presenting to emergency medical treatment with CAPS and who did not return to normal within 4 weeks following the event

sTable 7. Sensitivity analysis of rates of cannabis-associated psychotic symptoms (CAPS) in subsets of people who use cannabis

Sensitivity analysis	Subgroup	% CAPS (95% CI)	PWUC with CAPS	n PWUC included
Comparison between users of resin versus users of herbal cannabis when restricted to individuals residing in Denmark	Resin/Hash (in Denmark)	0.66 (0.46; 0.91)	36	5442
	Herbal cannabis (in Denmark)	0.37 (0.14; 0.81)	6	1617
Comparison between users of resin versus users of herbal cannabis when restricted to individuals not residing in Denmark	Resin/Hash (Denmark excluded)	0.21 (0.13; 0.32)	21	9956
	Herbal cannabis (Denmark excluded)	0.17 (0.14; 0.21)	100	58672

sTable 8. Reasons for not seeking emergency medical treatment following cannabis use in individuals from the United States

Reason for not seeking emergency medical treatment	Proportion (%)	n included
Knew would get better without help	36.23	69
Taken care of by friends	34.78	69
Thought it was not serious enough	26.09	69
Afraid of police	15.94	69
No insurance	5.8	69
Could not afford it	5.8	69
Friends told not to call for emergency medical treatment	2.9	69
No access to EMT	2.9	69
Taken care of by on-site first aid or medical care	0	69
Was alone and not capable of calling for help	0	69

Note. Data analysed from Global Drug Survey 2018, including only participants from the United States who indicated to have thought about seeking emergency medical treatment following the use of cannabis but did not end up seeking treatment.

sTable 9. Characterization of people who use cannabis and cannabis-associated psychotic symptoms (CAPS)

Characterization	Category	PWUC with last year CAPS: n (%)	PWUC with lifetime CAPS: n (%)
type of cannabis used before CAPS	edibles group	11 (6 %)	
	high potency herbal	78 (44 %)	219 (56 %)
	keif group	8 (4 %)	
	normal herbal	35 (20 %)	112 (29 %)
	oil group	4 (2 %)	12 (3 %)
	resin	42 (24 %)	47 (12 %)
other drug use before CAPS	alcohol	41 (21 %)	87 (23 %)
	amphetamine	10 (5 %)	11 (3 %)
	cocaine	7 (4 %)	7 (2 %)
	energy drink	1 (1 %)	4 (1 %)
	MDMA	20 (10 %)	11 (3 %)
	nothing	84 (43 %)	207 (54 %)
	other*	24 (12 %)	36 (9 %)
	tobacco	10 (5 %)	22 (6 %)
alcohol use before CAPS	between 1 and 5 drinks	45 (26 %)	
	between 10 and 15 drinks	2 (1 %)	
	between 5 and 10 drinks	10 (6 %)	
	more than 15 drinks	5 (3 %)	

	no drinks	110 (64 %)	
amount of cannabis used before CAPS	1g or less	201 (84 %)	
	between 1g and 2g	11 (5 %)	
	between 2g and 4g	13 (5 %)	
	more than 4g	13 (5 %)	
hospital admission following CAPS	no	178 (64 %)	225 (57 %)
	yes	99 (36 %)	168 (43 %)
time to recovery following CAPS	>4 weeks	47 (21 %)	56 (16 %)
	1 day or less	128 (56 %)	246 (69 %)
	1-2 days	33 (14 %)	31 (9 %)
	1-2 weeks	11 (5 %)	10 (3 %)
	2-4 weeks	10 (4 %)	15 (4 %)
Reductions in cannabis use following CAPS	no	119 (43 %)	160 (40 %)
	yes	160 (57 %)	239 (60 %)

Note. Sample restricted to people who use cannabis (PWUC) reporting cannabis-associated psychotic symptoms (CAPS) leading to emergency medical treatment. In the subsample of PWUC reporting lifetime occurrence of CAPS (cf. column 'PWUC with lifetime CAPS'), empty rows indicate that the corresponding variable was not assessed in this subset of PWUC

sTable 10. Subgroup analysis: Percentage of individuals requiring hospital admission as a result of cannabis-associated psychotic symptoms (CAPS)

31.48% (age 21 or younger)	42.86% (older than 21 years of age)	*p*=0.054
32.98% (female)	37.14% (male)	*p*=0.5
29.19% (less than one week to recover from CAPS)	54.41% (more than one week to recover from CAPS)	*p*=0.0003
31.54% (CAPS not involving hallucinations)	40.62% (CAPS involving hallucinations)	*p*=0.12
37.93% (CAPS not involving paranoia)	35.48% (CAPS involving paranoia)	*p*=0.79
26.32% (no lifetime diagnosis of psychosis)	76.32% (lifetime diagnosis of psychosis)	*p*=4.3e-08
38.10% (use of herbal cannabis before the occurrence of CAPS)	36.63 (use of high potency cannabis before the occurrence of CAPS)	*p*=0.82

sReferences

1. Jouanjus E, Leymarie F, Tubery M, Lapeyre-Mestre M. Cannabis-related hospitalizations: unexpected serious events identified through hospital databases. *Br J Clin Pharmacol*. 2011;71(5):758-765. doi:10.1111/j.1365-2125.2010.03897.x
2. Schmid Y, Scholz I, Mueller L, et al. Emergency department presentations related to acute toxicity following recreational use of cannabis products in Switzerland. *Drug Alcohol Depend*. 2020;206:107726. doi:10.1016/j.drugalcdep.2019.107726
3. Winstock A, Lynskey M, Borschmann R, Waldron J. Risk of emergency medical treatment following consumption of cannabis or synthetic cannabinoids in a large global sample. *J Psychopharmacol*. 2015;29(6):698-703. doi:10.1177/0269881115574493
4. Arendt M, Rosenberg R, Foldager L, Perto G, Munk-Jørgensen P. Cannabis-induced psychosis and subsequent schizophrenia-spectrum disorders: follow-up study of 535 incident cases. *Br J Psychiatry*. 2005;187(6):510-515. doi:10.1192/bjp.187.6.510
5. Hjorthøj C, Larsen MO, Starzer MSK, Nordentoft M. Annual incidence of cannabis-induced psychosis, other substance-induced psychoses and dually diagnosed schizophrenia and cannabis use disorder in Denmark from 1994 to 2016.

Psychol Med. Published online December 16, 2019:1-6. doi:10.1017/S0033291719003532

6. Winstock A, Barratt M, Ferris J. Global drug survey. *MixMag*. 2012;251:68-73.
7. Barratt MJ, Ferris JA, Zahnow R, Palamar JJ, Maier LJ, Winstock AR. Moving on From Representativeness: Testing the Utility of the Global Drug Survey. *Subst Abus Res Treat*. 2017;11:117822181771639. doi:10.1177/1178221817716391
8. Winstock AR, Barratt MJ. The 12-month prevalence and nature of adverse experiences resulting in emergency medical presentations associated with the use of synthetic cannabinoid products. *Hum Psychopharmacol Clin Exp*. 2013;28(4):390-393. doi:10.1002/hup.2292
9. Pearson NT, Berry JH. Cannabis and psychosis through the lens of DSM-5. *Int J Environ Res Public Health*. 2019;16(21):4149. doi:10.3390/ijerph16214149
10. Kelleher I, Harley M, Murtagh A, Cannon M. Are screening instruments valid for psychotic-like experiences? A validation study of screening questions for psychotic-like experiences using In-depth clinical interview. *Schizophr Bull*. 2011;37(2):362-369. doi:10.1093/schbul/sbp057
11. Potter DJ, Hammond K, Tuffnell S, Walker C, Di Forti M. Potency of Δ 9 -tetrahydrocannabinol and other cannabinoids in cannabis in England in 2016: Implications for public health and pharmacology. *Drug Test Anal*. 2018;10(4):628-635. doi:10.1002/dta.2368
12. Freeman TP, Morgan CJA, Hindocha C, Schafer G, Das RK, Curran HV. Just say ‘know’: how do cannabinoid concentrations influence users’ estimates of cannabis potency and the amount they roll in joints? *Addiction*. 2014;109(10):1686-1694. doi:10.1111/add.12634
13. van der Pol P, Liebregts N, de Graaf R, Korf DJ, van den Brink W, van Laar M. Validation of self-reported cannabis dose and potency: an ecological study. *Addiction*. 2013;108(10):1801-1808. doi:10.1111/add.12226
14. WHO. Body Mass Index standards.
15. Nakazawa M, Nakazawa MM. Package ‘fmsb.’ Published online 2019.
16. Rømer Thomsen K, Lindholst C, Thylstrup B, et al. Changes in the composition of cannabis from 2000–2017 in Denmark: Analysis of confiscated samples of cannabis resin. *Exp Clin Psychopharmacol*. 2019;27(4):402-411. doi:10.1037/pha0000303
17. Chandra S, Radwan MM, Majumdar CG, Church JC, Freeman TP, ElSohly MA. New trends in cannabis potency in USA and Europe during the last decade (2008–2017). *Eur Arch Psychiatry Clin Neurosci*. 2019;269(1):5-15. doi:10.1007/s00406-019-00983-5
18. Chan GCK, Hall W, Freeman TP, Ferris J, Kelly AB, Winstock A. User characteristics and effect profile of Butane Hash Oil: An extremely high-potency cannabis concentrate. *Drug Alcohol Depend*. 2017;178:32-38. doi:10.1016/j.drugalcdep.2017.04.014
19. Sarah Kliff. Emergency rooms are monopolies. Patients pay the price. Published 2017. Accessed May 20, 2020. https://www.vox.com/health-care/2017/12/4/16679686/emergency-room-facility-fee-monopolies
20. Hamilton I, Winstock A. Cannabis isn’t the health problem – the tobacco people mix with it is. *Conversat*. Published online 2017. https://theconversation.com/cannabis-isnt-the-health-problem-the-tobacco-people-mix-with-it-is-77067
21. Hindocha C, Freeman TP, Ferris JA, Lynskey MT, Winstock AR. No smoke without tobacco: A Global overview of cannabis and tobacco routes of administration and their association with intention to quit. *Front Psychiatry*. 2016;7. doi:10.3389/fpsyt.2016.00104
22. Winstock AR, Wolff K, Ramsey J. 4-MTA: a new synthetic drug on the dance scene. *Drug Alcohol Depend*. 2002;67(2):111-115. doi:10.1016/S0376-8716(02)00018-2
23. Winstock AR, Griffiths P, Stewart D. Drugs and the dance music scene: a survey of current drug use patterns among a sample of dance music enthusiasts in the UK. *Drug Alcohol Depend*. 2001;64(1):9-17. doi:10.1016/S0376-8716(00)00215-5
24. Winstock AR, Barratt MJ. Synthetic cannabis: A comparison of patterns of use and effect profile with natural cannabis in a large global sample. *Drug Alcohol Depend*. 2013;131(1-2):106-111. doi:10.1016/j.drugalcdep.2012.12.011
